# Underwater Multi-Target Tracking and Behavioral Rhythm Analysis of Chinese Giant Salamander Based on TransTrack-OC-SORT

**DOI:** 10.3390/ani16101479

**Published:** 2026-05-12

**Authors:** Nanqing Sun, Xinyao Yang, Mokai Xie, Haotian Qian, Junyi Chen

**Affiliations:** 1 School of Intelligent Science and Engineering, Hubei Minzu University, Enshi 445000, China; 202430357@hbmzu.edu.cn (N.S.); xyy0138@163.com (X.Y.); 202530360@hbmzu.edu.cn (M.X.); stromqdd@163.com (H.Q.); 2 Key Laboratory of Green Manufacturing of Super-Light Elastomer Materials, State Ethnic Affairs Commission, Hubei Minzu University, Enshi 445000, China

**Keywords:** Chinese giant salamander, deep learning, behavioral monitoring, transform, BIOU

## Abstract

To address the challenges posed by traditional tracking algorithms in adapting to complex underwater environments characterized by nonlinear motion, drastic morphological changes, mimicry camouflage, and frequent occlusions in wild Chinese giant salamanders, this study proposes a multi-object tracking and behavior analysis method based on the TransTrack-OC-SORT algorithm. The algorithm employs a dual-branch Transformer motion predictor to replace linear Kalman filtering, effectively capturing nonlinear motion patterns such as velocity changes and directional turns exhibited by salamanders. Simultaneously, it introduces the BIOU matching metric, which integrates center distance and aspect ratio penalties with overlap degree, thereby enhancing the robustness of associations in scenarios involving occlusion and mimicry camouflage. The results indicate that the multi-object tracking accuracy (MOTA) based on the TransTrack-OC-SORT algorithm reaches 80.9%, while the identity preservation metric (IDF1) achieves 83.7%. Quantitative behavioral analysis based on continuous trajectory data obtained from this algorithm revealed significant diurnal behavioral rhythms in the Chinese giant salamander. During the daytime, stationary behavior accounted for 95.5% of the total behavioral duration, while swimming for ventilation and foraging behaviors constituted only 4.1% and 0.4%, respectively. At night, the salamander’s behavioral patterns underwent fundamental changes; although stationary behavior remained dominant (approximately 72.6%), the proportions of swimming for ventilation and foraging behaviors significantly increased to 15.1% and 12.3% of the nocturnal period, respectively. These findings enhance our understanding of the salamander’s ecological habits from the perspectives of visual adaptation, energy allocation, and predation strategies. This study provides a reliable technical tool for non-invasive behavioral monitoring and rhythm research in endangered amphibians.

## Simple Summary

The wild Chinese giant salamander is an endangered species. Its nocturnal habits, preference for hiding in rock crevices, and body coloration that closely resembles riverbed rocks pose significant challenges for scientists attempting to continuously observe and record its behavior. To accurately track the movement trajectories of giant salamanders and analyze their daily activity patterns, this study developed a novel computer vision algorithm named TransTrack-OC-SORT. This algorithm effectively predicts salamanders’ irregular swimming paths and maintains stable tracking even when the animals are occluded or camouflaged. The experimental results indicate that this innovative approach significantly outperforms existing mainstream techniques such as SORT, DeepSORT, and ByteTrack, achieving a tracking accuracy of 80.9% MOTA and an identity recognition capability of 83.7% IDF1, while reducing the number of identity switches to just 12 instances. Through the continuous analysis of acquired movement trajectories and quantitative statistics, we identified that the Chinese giant salamander exhibits pronounced diurnal behavioral rhythms. Specifically, it remains nearly motionless during the day, with stationary behavior accounting for 95.5% of its activity. In contrast, it demonstrates relatively frequent swimming, surfacing for air, and foraging at night, with swimming and surfacing increasing to 15.1% and foraging rising to 12.3%. This discovery enhances our understanding of the living habits of the Chinese giant salamander. The methodology employed demonstrates universality in its technical architecture. The core Transformer-based motion prediction module can be utilized to learn movement patterns of various wildlife species, while the BIOU association metric facilitates the recalibration of prior parameters based on the morphological characteristics of target species. Consequently, this approach can be readily extended to non-contact behavioral monitoring of other wildlife that face similar surveillance challenges, such as strong concealment capabilities or complex activity environments, thereby contributing to efforts in biodiversity conservation.

## 1. Introduction

The Chinese giant salamander, an endangered amphibian species, relies on long-term and precise behavioral monitoring data to effectively conserve its wild populations and habitats [1,2,3,4]. Continuous behavioral trajectories for this species contain critical biological and ecological information [5,6,7]. For example, courtship migration paths during the breeding season can reflect population vitality, while abnormal surfacing frequency or prolonged immobility may indicate water hypoxia or disease infection. Therefore, establishing a high-precision trajectory tracking system is a crucial tool for transitioning from population surveys to refined behavioral ecology research.

In recent years, advancements in computer vision technologies, particularly those based on deep learning for object detection and tracking, have paved the way for non-invasive trajectory tracking and monitoring of wildlife [8,9,10,11,12]. Chen Hao et al. proposed YOLO-OCS, a feature fusion-based algorithm designed for dense pedestrian detection and tracking, which aims to overcome significant challenges such as inadequate detection accuracy and partial target occlusion in traffic scenarios [13]. Diogo Ferreira et al. developed an autonomous vision-based system for tracking and following moving targets, specifically tailored for unmanned aerial vehicles (UAVs) and leveraging multi-target information [14]. Additionally, Shuai Wang introduced a joint detection and tracking method named Visibility-Guided Tracking for Multiple Object Tracking (VGT-MOT), which addresses the challenges of accurately estimating object positions amidst rapid changes in camera or object motion [15]. However, directly applying existing Multiple Object Tracking (MOT) algorithms to the tracking of underwater giant salamanders presents significant challenges. First, the skin color of giant salamanders (e.g., brown, dark gray) closely resembles the rocks and fallen leaves found in streams, leading to extremely low target-background contrast and substantial fluctuations in detection confidence. Second, when crawling or preying, giant salamanders disturb bottom sediments, creating transient turbid areas that severely disrupt visual target features. Third, uneven lighting in forest streams, reflections from the water surface, and drastic variations in diurnal light ratios introduce considerable noise into feature extraction and appearance modeling. Fourth, giant salamanders are primarily nocturnal, and low-light conditions or near-infrared supplementary lighting can result in blurred image details and loss of color information. Finally, their frequent habitation in rock crevices or aquatic vegetation leads to recurrent partial occlusions. To address these challenges, including nonlinear motion, drastic feature variations, mimicry camouflage, and long-term occlusion, existing algorithms such as SORT [16,17,18], DeepSORT [19,20,21], and their variants like ByteTrack [22,23,24] predominantly rely on constant velocity motion models and simplistic appearance feature matching, which are inadequate for effective resolution.

The primary objective of this study is to develop an algorithmic framework that improves multi-object tracking accuracy and enhances identity preservation robustness, addressing prevalent challenges in underwater giant salamander tracking, such as nonlinear motion, drastic morphological changes, mimicry camouflage, and frequent occlusions. To achieve this, we propose TransTrack-OC-SORT, an algorithm that integrates biological knowledge with environmentally adaptive design. This approach aims to ensure stable and continuous tracking of individual giant salamanders in complex underwater environments, thereby providing reliable technical support for quantitative behavioral ecology analysis.

## 2. Materials and Methods

### 2.1. Study Area and Hardware Deployment

**Figure 1:**
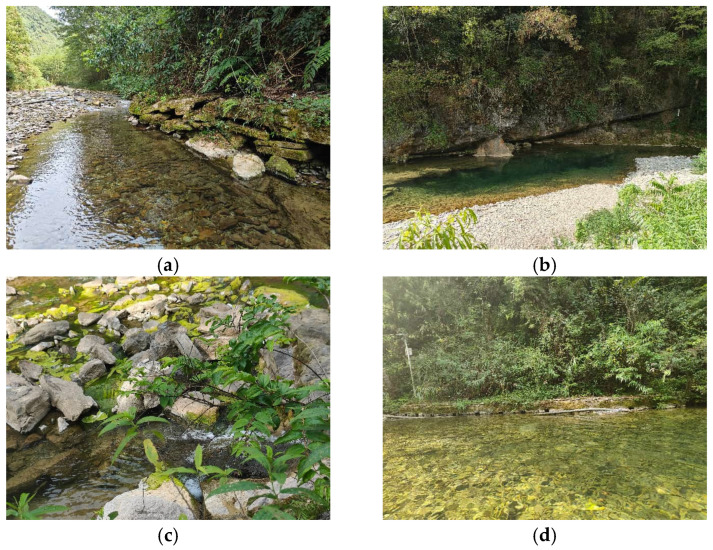
Monitoring plots in different scenarios. (**a**) shoal. (**b**) deep pool. (**c**) backwater area. (**d**) cave.

**Figure 2:**
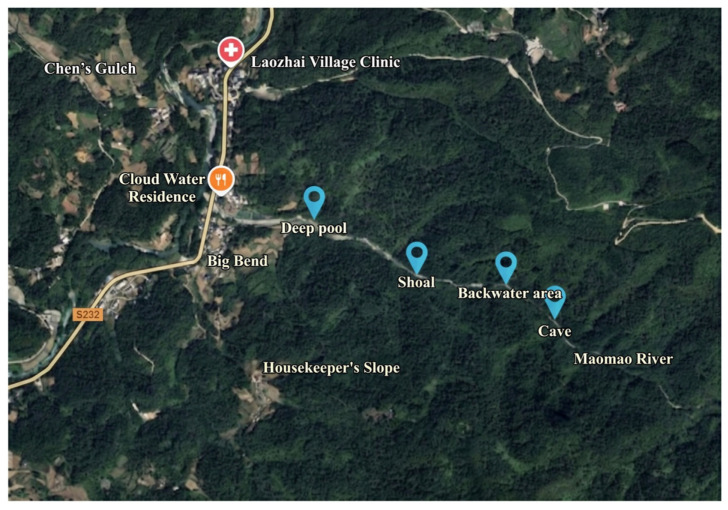
Location map of the four monitoring sections.

This study was conducted in the Zhongjian River Chinese Giant Salamander National Nature Reserve (29–30° N, 108–109° E) located in Xianfeng County, Hubei Province. Nestled in the Wuling Mountain area, the reserve is characterized by unique karst landforms and a subtropical monsoon climate, which together provide an excellent habitat and breeding grounds for the Chinese giant salamander. To comprehensively capture the behavioral patterns of the Chinese giant salamander (*Andrias davidianus*) in its natural environment, we established four fixed monitoring transects (Figure 1) based on the following selection criteria: (1) During the initial establishment of the reserve, the management committee delineated the core area, and numerous news reports have documented the presence of wild giant salamanders in this region, allowing us to designate it as an observation site; (2) Preliminary field surveys conducted from September to November 2024 recorded the frequency of nocturnal activities and daytime concealment behaviors of salamanders, leading us to refine the core area identified in criterion 1 to specific river sections; (3) Within these river sections, we selected sites that encompass diverse habitat types, including deep pools, shallow shoals, backwater areas, and cave entrances, to ensure ecological diversity and the completeness of the behavioral monitoring data. The straight-line distances between monitoring sites along the river channel are approximately 300 m between the riffle and pool, 210 m between the riffle and backwater area, and 150 m between the backwater area and cave. This spacing ensures relative independence among sites, thereby avoiding repeated counts of the same giant salamander individual within a short period, while simultaneously forming a spatially continuous monitoring network within the core zone of the reserve (Figure 2).

Data collection was conducted using a high-definition network camera array (Equipment model: TL-IPC6128-EZ, Manufacturer name: TP-Link, City: Shenzhen Country: China) deployed along the shoreline. The cameras operated continuously, 24 h a day, at a frame rate of 30 FPS and a resolution of 4512 × 2512. Equipped with quad-pixel binning technology, these cameras effectively meet the requirements for nighttime monitoring. The collection period spanned from December 2024 to December 2025, comprehensively documenting natural environmental conditions across various daylight periods (daytime, dusk, night), weather conditions, and water levels (dry season, flood season). Over 500 h of raw monitoring video were accumulated, and detailed camera configurations are presented in Table 1 below.

### 2.2. Data Collection and Image Annotation

**Figure 3:**
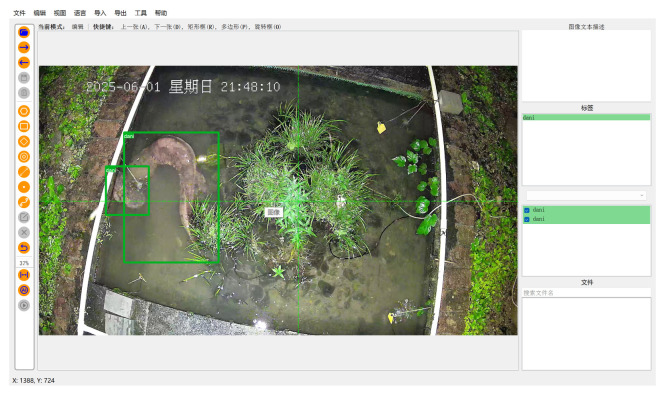
Labeling giant salamander images using X-anylabeling. Note: The non-English term “星期日” in the figure translates to “Sunday”.

**Figure 4:**
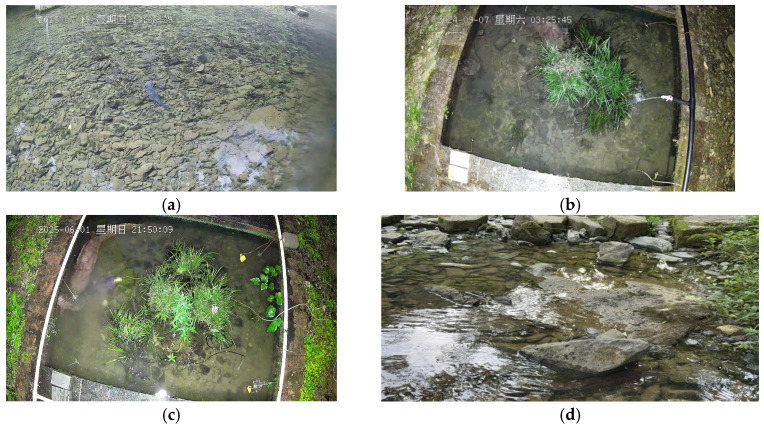
Image Dataset. (**a**) daytime. (**b**) nighttime. (**c**) turbid water. (**d**) frequent occlusion. Note: The non-English term “星期四” in the figure translates to “Thursday”. The non-English term “星期六” in the figure translates to “Saturday”. The non-English term “星期日” in the figure translates to “Sunday”.

Through technical screening and ecological discrimination of extensive raw video footage, we manually selected high-quality video clips that prominently feature identifiable activities of individual giant salamanders (Table 2). Subsequently, we employed X-anylabeling (v2.4.4) to accurately annotate the bounding boxes of all giant salamander individuals in each frame (Figure 3), assigning unique identity IDs to construct ground truth data suitable for supervised training and algorithm evaluation. The core dataset of this study comprises 45 independent video sequences, resulting in 13,545 annotated bounding boxes. To ensure a rigorous evaluation, the dataset was randomly divided into training, validation, and test sets in an 8:1:1 ratio. The test set is further subdivided into four subsets based on predominant challenges: daytime, nighttime, turbid water, and frequent occlusion (Figure 4), allowing for an analysis of algorithm robustness under specific challenging scenarios.

### 2.3. Multi-Object Tracking

**Figure 5:**
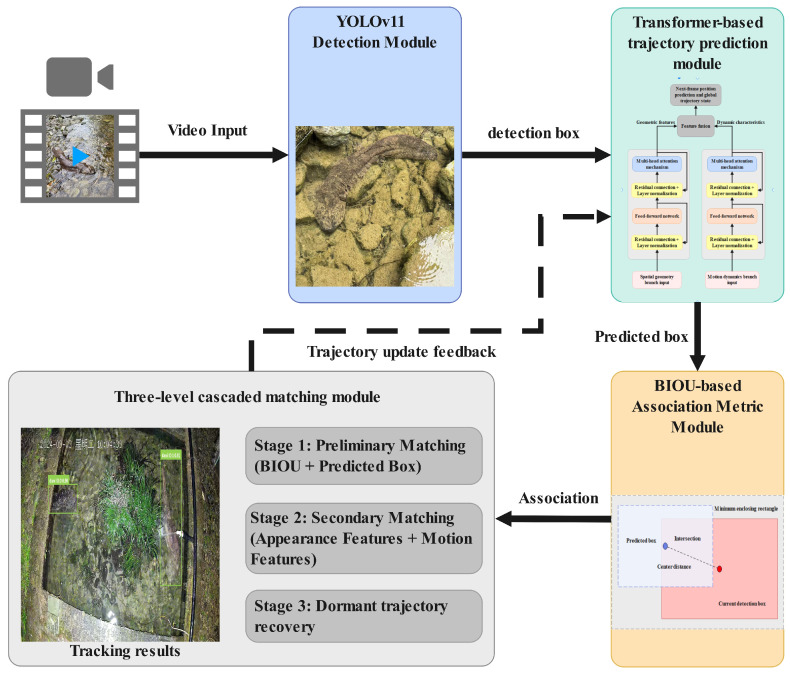
The framework of TransTrack-OC-SORT algorithm. Note: The non-English term “星期二” in the figure translates to “Tuesday”.

To address the specific challenges of tracking giant salamanders in complex underwater environments, this study proposes the TransTrack-OC-SORT algorithm (Figure 5), which consists of four core modules: detection, trajectory prediction, association metric, and cascade matching. The detection module utilizes YOLOv11, which has been fine-tuned on a self-constructed dataset, to generate target bounding boxes and confidence scores. The trajectory prediction module replaces the Kalman filter with a dual-branch Transformer, using historical trajectory state sequences as input to predict the position in the next frame. The association metric module introduces BIOU, enhancing tolerance to deformation and occlusion by incorporating penalties for center distance and aspect ratio based on IoU. Finally, the cascade matching module operates in three stages: it first performs rapid matching based on BIOU, then refines this matching by integrating appearance and motion features, and finally reactivates disappeared targets and recovers trajectories using a dormant trajectory buffer.

#### 2.3.1. Object Detection

Given the concealment of giant salamanders in complex underwater environments, the target detection module must ensure stable object capture under low-contrast and highly camouflaged conditions. This project selects YOLOv11 as the base detector, based on several key considerations. First, YOLOv11 achieves an optimal balance between detection accuracy and inference speed, satisfying the real-time requirements for prolonged field monitoring. Second, its feature pyramid and attention mechanism enhance the capture of subtle visual features of giant salamanders amidst significant background interference, thereby providing high-quality input for target tracking.

To address the unique characteristics of the Chinese giant salamander’s flattened body shape and muted coloration, we adapted and fine-tuned YOLOv11 in two specific aspects, utilizing our previously established dataset.

(1) Data Augmentation Strategy

To address the challenges posed by uneven underwater lighting, turbid water, and low-illumination nighttime scenarios, we introduce mosaic augmentation and random flipping as strategies to enhance the model’s robustness against variations in lighting and disturbances in water quality. This approach exposes the model to a diverse range of degradation conditions during the training phase, thereby improving its generalization capability for real-world deployment.

(2) Adaptive Adjustment of Confidence Threshold

Given that detection confidence scores tend to be lower in nighttime and turbid scenarios, setting an excessively high threshold may result in numerous missed detections. Our tracking framework employs a dynamic confidence strategy: when the environmental perception module identifies challenging scenarios, it appropriately lowers the minimum confidence threshold for detection outputs, thereby retaining more low-confidence candidate bounding boxes. These candidates are subsequently subjected to secondary discrimination through BIOU association metrics and a three-stage cascaded matching process, leveraging the tracker’s robustness to compensate for the detector’s limitations.

#### 2.3.2. Transformer-Based Trajectory Prediction

One of the core tasks of target tracking algorithms is to accurately predict the future motion state of the target, thereby facilitating data association in the detection of the current frame. The movement of the giant salamander in water does not follow a uniform linear trajectory; instead, it exhibits typical nonlinear behavioral patterns, including stationary periods, bursts of activity, turning, and oscillation. To address this complexity, a lightweight TrajectoryTransformer module has been designed. This module capitalizes on the robust sequence modeling and long-range dependency capturing capabilities of the Transformer architecture, with the aim of learning the intrinsic motion patterns from the target’s historical trajectory and performing nonlinear extrapolation of future states.

To achieve effective sequence learning, the primary task is to convert the target’s motion trajectory into temporal features that are suitable for processing by Transformers. For each active tracking trajectory, we extract the most recent *L* = 10 consecutive frames of states (where the value of *L* is determined through sensitivity experiments on the validation set), thereby forming a historical state sequence *H* = [*h*_*t*−*L*_, …, *h*_*t*−1_]. Each state *h_i_* at time step i is represented as a 6-dimensional feature vector.

(1) $$h_{i}=\left[x_{i},\;y_{i},{\;w}_{i},{\;h}_{i},{\;\Delta x}_{i},\;{\Delta y}_{i}\right]$$

In the equation, (*x_i_*, *y_i_*) represents the normalized image coordinates of the target bounding box center; (*w_i_*, *h_i_*) denotes the normalized width and height of the target bounding box. (Δ*x_i_*, Δ*y_i_*) indicates the normalized displacement of the bounding box center between the current frame and the previous frame, which is a discrete approximation of the instantaneous velocity.

This representation simultaneously incorporates the target’s absolute spatial position, scale information, and instantaneous motion trends, providing the model with comprehensive motion context.

**Figure 6:**
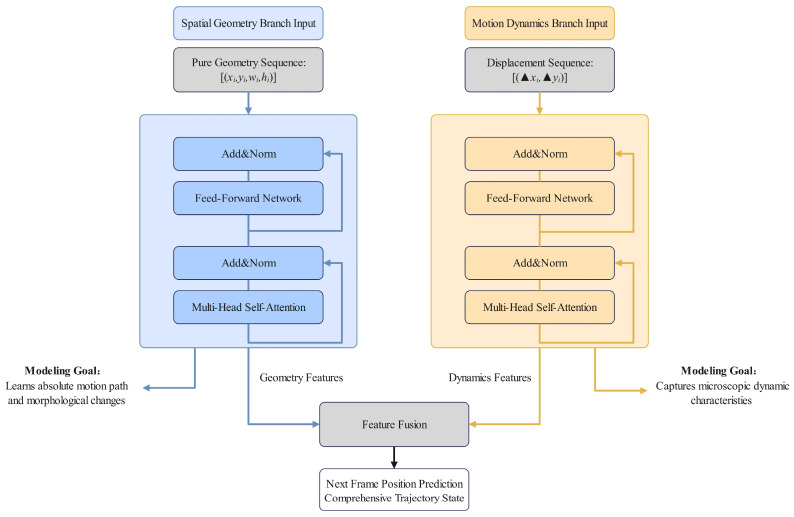
Dual-branch Transformer Encoder Architecture.

To achieve finer-grained decoupling and modeling of trajectory information, we designed a unique dual-branch Transformer encoder architecture (Figure 6). It should be noted that although Equation (1) defines all 6-dimensional states within the same vector, the actual input data for the two branches are strictly separated: the geometric branch only takes (*x_i_*, *y_i_*, *w_i_*, *h_i_*) as input, while the motion dynamics branch exclusively processes (Δ*x_i_*, Δ*y_i_*). Although displacement is mathematically the first-order difference in position, decoding them separately allows the geometric branch to focus on learning macro-level spatial trajectories and morphological changes, while the motion branch concentrates on capturing micro-level dynamic features such as instantaneous velocity variations and acceleration. This approach enables complementary motion representations at different temporal granularities.

The left branch represents the spatial geometry component, which processes pure geometric sequences represented as [(*x_i_*, *y_i_*, *w_i_*, *h_i_*)]. The primary objective of this branch is to learn the absolute motion paths and morphological change patterns of the target within the image space. It effectively captures the macro-level trajectory of the giant salamander as it meanders along the riverbed, as well as the periodic scale variations in its body swaying in response to the water flow. Conversely, the right branch is dedicated to motion dynamics, utilizing displacement sequences denoted as [(Δ*x_i_*, Δ*y_i_*)]. This branch aims to extract micro-level dynamic characteristic parameters related to the target’s movement, including acceleration, abrupt changes in motion direction, and transition patterns between stationary states and initiation. This analysis is essential for predicting behaviors such as the giant salamander’s sudden launch into an attack from concealment or its rapid escape when startled.

The two branches share identical foundational Transformer encoder configurations, consisting of 2 encoder layers, 4 attention heads, and a feed-forward network dimension of 128. However, they possess completely independent parameters. This design facilitates specialized optimization for each branch’s specific input modality, thereby enhancing feature extraction effectiveness within their respective domains.

At the same time, the Transformer encoders of the two branches output corresponding high-dimensional temporal features respectively. We take the feature vectors *F_geom_* and *F_motion_* from the two branches at frame *t* − 1, and concatenate them to form a fused feature *F_fusion_*.

The fused feature *F_fusion_* is then fed into a lightweight multi-layer perceptron for decoding. The output of the MLP is a 4-dimensional vector representing the predicted bounding box offsets:

(2) $$\left[\Delta \hat{x},\;\Delta \hat{y},\;\Delta \hat{w},\;\Delta \hat{h}\right]=\mathrm{MLP}$$

Ultimately, the predicted bounding box ${\hat{b}}_{t}$ for the current frame (frame *t*) is obtained by adding the predicted offset to the estimated bounding box *b*_*t*−1_ from the previous frame:

(3) $${\hat{b}}_{t}\;=\;b_{t-1}\;+\;\left[\Delta \hat{x},\;\Delta \hat{y},\;\Delta \hat{w},\;\Delta \hat{h}\right]$$

To make the predictions more aligned with the physiological reality of the Chinese giant salamander, we incorporated biological constraints into the model training. Although the salamander’s burst swimming speed is fast, it has a physiological upper limit. We transformed this prior knowledge into a regularization term in the training loss function.

Let the calibration coefficient from image pixels to real-world space be *k* (meters/pixel), and the instantaneous velocity predicted by the model is

(4) $$v_{\mathrm{pred}}=k\sqrt{{\left(\Delta \hat{x}\right)}^{2}+{\left(\Delta \hat{y}\right)}^{2}}$$

This paper defines the velocity constraint loss *L_velocity_* as

(5) $$L_{\mathrm{velocity}}=\lambda \cdot \max{\left(0,{\;v}_{\mathrm{pred}}-v_{\max}\right)}^{2}$$

In the equation, *v_max_* = 2.0 m/s represents the preset maximum reasonable velocity of the giant salamander, and *λ* denotes the equilibrium weight coefficient.

This loss term, together with the smooth loss *L_reg_* between the predicted box and the ground truth box, constitutes the total loss *L_total_*, expressed by the formula

(6) $$L_{\mathrm{total}}=L_{\mathrm{reg}}+L_{\mathrm{velocity}}$$

It guides the model to generate biomechanically plausible trajectories while pursuing prediction accuracy.

#### 2.3.3. BIOU Correlation Metric

To address the limitations of traditional Intersection over Union (IoU) metrics in complex underwater environments, we propose a novel association metric termed BIOU. BIOU incorporates an additional geometric consistency constraint that enhances the overlap area measurement. The calculation formula for BIOU is as follows:

(7) $$\mathrm{BIOU}\;=\;\mathrm{IoU}-\frac{\rho^{2}\left(b_{p},b_{d}\right)}{c^{2}}-\alpha v$$

In the equation, *ρ*^2^(*b_p_*, *b_d_*) represents the squared Euclidean distance between the center points of the predicted box *b_p_* and the detection box *b_d_*. c denotes the diagonal length of the smallest enclosing region covering both *b_p_* and *b_d_*. $v\;=\;\frac{4}{\pi^{2}}{\left(\mathrm{arc}\tan\frac{w_{p}}{h_{p}}-\mathrm{arc}\tan\frac{w_{d}}{h_{d}}\right)}^{2}$ is a term measuring the consistency of aspect ratios. *α* represents the balancing weight.

**Figure 7:**
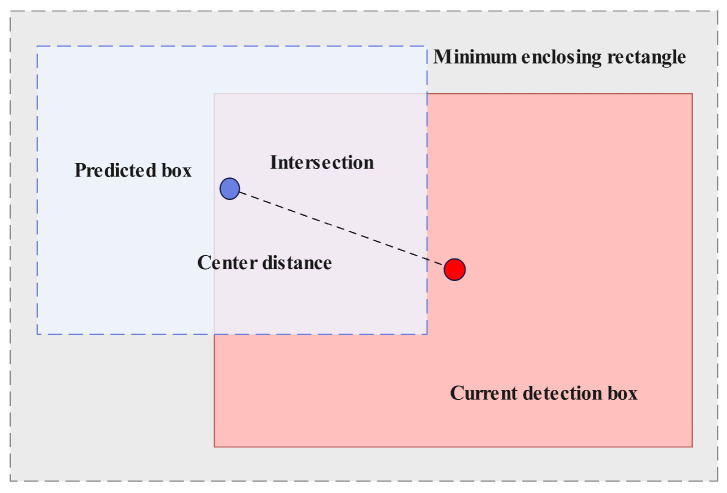
BIOU schematic diagram.

The introduction of the aspect ratio penalty term in this paper is not solely based on geometric considerations; rather, it is grounded in rigorous biological statistics. We conducted a morphological analysis of 13,545 giant salamander samples from the dataset and found that their length-to-width ratio during normal swimming consistently remained within the range of [1:4, 1:8], with only brief extreme values occurring during sharp turns. BIOU (Figure 7) employs this prior distribution to dynamically adjust the penalty weight: when the aspect ratio of a predicted bounding box significantly deviates from this biologically statistical range (for example, when misidentified as a square-shaped rock), the penalty term increases exponentially, thus mathematically eliminating false detections that do not conform to the anatomical characteristics of giant salamanders.

The role of this aspect ratio prior is not to identify species but to serve as a geometric constraint during the data association stage, effectively filtering out false detections where the shapes of bounding boxes significantly deviate from the body morphology of giant salamanders. The differentiation of giant salamanders from other aquatic organisms is achieved through the YOLOv11 detector, which is trained on specialized datasets. Ecologically, giant salamanders are apex predators within stream ecosystems. In the monitored habitats of this study—riffles, pools, backwaters, and caves—few other species exhibit comparable body size and morphology. Co-occurring fish species typically display slender bodies, with adult lengths usually under 15 cm, resulting in a clear separation in aspect ratios from the [1:4, 1:8] range. Other amphibians and crustaceans also exhibit markedly distinct body shapes. Consequently, this biological constraint does not introduce additional identity uncertainty due to species confusion.

#### 2.3.4. Three-Stage Cascaded Matching and Feature Fusion

**Figure 8:**
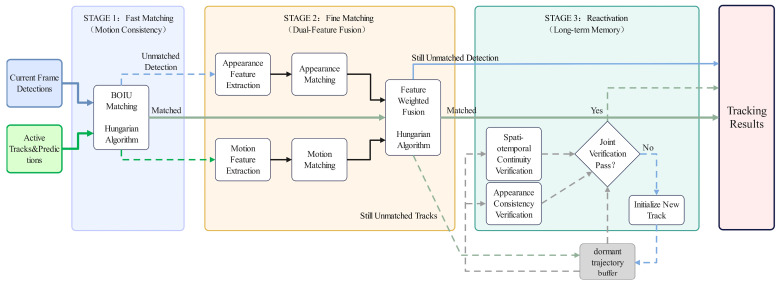
Three-stage cascaded data association.

After obtaining the object detection results for the current frame and implementing Transformer-based trajectory prediction, we systematically address association problems of varying complexity levels (Figure 8) following the principle of progressing from simple to complex and from coarse to fine. This hierarchical approach ensures computational efficiency while maximizing both tracking continuity and accuracy.

(1) **Phase 1: Rapid Matching Based on Motion Consistency**

This stage aims to efficiently associate targets with smooth motion trajectories and high visibility. The TrajectoryTransformer module generates predicted bounding boxes in the current frame for each active trajectory. Subsequently, a BIOU similarity matrix, as defined in Equation (7), is computed between all predicted boxes and all detection boxes in the current frame. The BIOU metric integrates geometric overlap, center distance, and aspect ratio, demonstrating greater tolerance to localization jitter and minor deformations compared to traditional IoU. The Hungarian algorithm is employed to solve this cost matrix for global optimal matching, completing the preliminary association. To adapt to environmental variations, this phase employs a dynamic matching threshold, *τ_biou_*: it is set to 0.3 during daytime and occluded scenarios while being appropriately reduced to 0.2 for nighttime and turbid water conditions to accommodate more uncertain matching associations and prevent early trajectory loss due to degraded image quality. These thresholds were determined through grid search on the validation set (Section 3.3), with Multiple Object Tracking Accuracy (MOTA) as the optimization metric, selecting the parameter combination that yields optimal tracking performance across scenario subsets. For practical all-weather deployment, a lightweight online discrimination module based on image statistics can be incorporated—for instance, using frame average brightness to distinguish day/night conditions or employing pixel variance and edge density to indicate water turbidity—enabling automatic threshold switching without manual annotation.

(2) **Phase 2: Fine-grained Matching Based on Dual Feature Fusion**

Trajectories and detection boxes that fail to match in the initial stage often encounter significant occlusion, strong reflections, or similarities in appearance between targets. To mitigate these challenges, this stage introduces more comprehensive feature representations for fine-grained re-identification. For every trajectory and detection box that requires matching, two types of features are extracted in parallel.

Appearance features: A lightweight convolutional neural network pre-trained on a ReID dataset is employed to extract high-dimensional deep feature vectors (*f_a_*) from the target crop regions. The cosine similarity (*S_a_*) between the historical appearance features of the trajectory and the appearance features of the current detection box is calculated. Cosine similarity is insensitive to scale variations in feature vectors, enabling effective measurement of appearance pattern similarity.

Motion Characteristics: For the predicted trajectory bounding box and the detection box to be matched, compute the dense optical flow field between corresponding image regions in adjacent frames. Quantize the angle of optical flow vectors into several intervals to construct an optical flow direction histogram, thereby characterizing the local motion pattern of the target. Calculate the Bhattacharyya coefficient between the historical motion pattern of the trajectory and the current motion pattern of the detection region as the motion similarity *S_m_*. The Bhattacharyya coefficient is suitable for histogram comparison and can effectively measure the consistency of motion distributions. Finally, perform weighted fusion of appearance and motion similarities to obtain the comprehensive similarity score S.

(8) $$S=\omega S_{a}+\left(1-\omega \right)S_{m}$$

In this paper, *ω* denotes the fusion weight for appearance and motion similarity, which is set to 0.7. This value indicates that appearance features serve as the primary association cues in most scenarios, while motion continuity offers supplementary discriminative information. The specific value of *ω* is established through parameter sensitivity analysis conducted on the validation set (see Section 3.3).

(3) **Phase 3: Reactivation of Dormant Trajectories Based on Long-Term Memory**

To address situations where the giant salamander may remain hidden in caves or be completely obscured for extended periods, it is classified as dormant and subsequently transferred into a separate dormant trajectory buffer. The trajectories within this buffer preserve their appearance features, motion model (Transformer state), and spatial contextual information prior to being obscured. When new detection boxes remain unmatched after the second stage, the algorithm compares them with the trajectories stored in the dormant buffer. The reactivation process is determined through a comprehensive decision-making procedure that necessitates the simultaneous fulfillment of the following conditions:

(1) Spatiotemporal continuity: The newly detected position aligns with the predicted location and motion direction at the time of trajectory loss, with the time interval falling within a reasonable range.
(2) The appearance characteristics of the new detection closely resemble the stored appearance memory of the dormant trajectory. The reactivation process is initiated only when the combined confidence surpasses a predetermined high threshold, thereby re-associating this detection box with the dormant trajectory and reintegrating it into the active trajectory set. This mechanism effectively addresses the problem of trajectory identity loss that can occur due to prolonged and total occlusion, facilitating coherent tracking throughout the entire lifecycle of target appearance, disappearance, and reappearance.

The aforementioned three-stage cascaded matching framework establishes a data association engine that balances efficiency, robustness, and long-term consistency through its progressive geometric feature memory association logic. This framework serves as the foundational guarantee for the TransTrack-OC-SORT algorithm, ensuring high tracking accuracy in complex environments.

#### 2.3.5. Software and Parameter Settings

All experiments were conducted on a workstation equipped with an NVIDIA GeForce RTX 4070 GPU, with Python 3.8 and PyTorch 1.10 as the software environment. During the model training phase, both the YOLOv11 detector and TrajectoryTransformer predictor were trained for 500 epochs with a batch size of 8 and an initial learning rate of 0.001 using the Adam optimizer. In the tracking inference phase, the BIOU dynamic matching threshold *τ_biou_* was set to 0.30 for daytime and occlusion scenarios and 0.20 for nighttime and turbid scenarios; the second-stage fusion weight *ω* was set to 0.70. The values of these parameters were determined through sensitivity analysis on the validation set (Section 3.3).

#### 2.3.6. Behavior Classification Based on Tracking Trajectories

To support the analysis of circadian behavioral rhythms, the continuous tracking trajectories produced by TransTrack-OC-SORT must be converted into discrete behavioral categories. This study employs a rule-based determination method that utilizes trajectory motion features, with the specific rules outlined as follows:

(1) Stationary Behavior: A target is classified as stationary when the inter-frame displacement (Δ*d*) of its bounding box center is less than 0.005, and the instantaneous velocity (*v*) is below 0.02 m/s over a continuous time window of at least 3 s.
(2) Ventilatory swimming behavior is identified when the inter-frame displacement (Δ*d*) of the target bounding box center exceeds 0.01 and the instantaneous velocity (*v*) is greater than 0.08 m/s, accompanied by body undulation.
(3) Foraging behavior: When the displacement velocity *v* is between 0.02 m/s and 0.08 m/s, and the movement direction changes by more than 60° within a 10 s window, it is identified as foraging.

## 3. Experimental Results and Analysis

### 3.1. Performance Comparison and Evaluation of Multiple Tracking Algorithms

We compared TransTrack-OC-SORT with mainstream algorithms such as DeepSORT, ByteTrack, and OC-SORT on our self-built giant salamander dataset. Table 3 presents the performance comparison results of these algorithms.

Comprehensive comparative experiments were conducted between TransTrack-OC-SORT and mainstream tracking algorithms using our self-constructed giant salamander dataset. As illustrated in Table 3, the proposed algorithm achieved optimal performance across multiple core metrics: its Multiple Object Tracking Accuracy (MOTA) reached 80.9%, which represents a 4.1% improvement over the second-best performing OC-SORT, and the Identity F1 Score (IDF1) reached 83.7%, indicating a 3.5% enhancement. More importantly, the algorithm demonstrated exceptional performance in tracking continuity and stability, with identity switches (IDs) and trajectory fragmentations (Frag) reduced to 12 and 10, respectively, marking significant improvements over baseline methods. The experimental results indicate that TransTrack-OC-SORT effectively balances precision, robustness, and real-time performance, with its overall performance significantly surpassing that of existing methods.

To empirically validate the advantages of the proposed Bounded-IoU (BIOU) association metric over existing generalized IoU variants, we conducted dedicated comparative experiments. Utilizing the confirmed optimal TransTrack-OC-SORT algorithm, we replaced only the similarity measurement method in its first-stage fast matching and evaluated performance on the SalamanderTrack test set, as well as various challenge subsets (see Table 4). The IoU metrics compared include standard IoU, Generalized IoU (GIoU), Distance-IoU (DIoU), and Complete IoU (CIoU).

As illustrated in Table 4, the evolution from the traditional Intersection over Union (IoU) to its enhanced variants—Generalized IoU (GIoU), Distance IoU (DIoU), and Complete IoU (CIoU)—shows a progressive incorporation of considerations for center distance and aspect ratio in the association metrics. Consequently, the tracking performance metrics, namely Multiple Object Tracking Accuracy (MOTA) and ID F1 Score (IDF1), exhibit incremental improvements, while the occurrences of identity switches (IDs) and trajectory fragments (Frag) decrease correspondingly. This trend underscores the necessity of integrating additional geometric constraints in complex scenarios. Following this evolutionary trajectory, the Bounding IoU (BIOU) metric introduced in this study achieves superior overall performance, with both MOTA and IDF1 surpassing those of the conventional CIoU metric. Notably, the Frag metric, which assesses trajectory continuity, declines to as few as 10 instances. The experimental results indicate that task-specific designs for association metrics can yield more precise matching and more stable tracking performance compared to methods that prioritize generality.

### 3.2. Ablation Experiment

To validate the effectiveness of each improved module, we conducted ablation experiments based on OC-SORT, with the results shown in Table 5.

The results of the ablation experiment are presented in Table 5. In the OC-SORT baseline, the introduction of the dual-branch Transformer prediction module resulted in a decrease in the number of identity switches (IDs) from 18 to 16, reflecting an approximate reduction of 11%. Additionally, trajectory fragments (Frag) decreased from 15 to 13, indicating a reduction of about 13%. This suggests that accurate modeling of the salamander’s nonlinear motion effectively mitigates trajectory breaks caused by prediction deviations. Furthermore, the introduction of the BIOU association metric led to a decrease in trajectory fragments (Frag) from 15 to 12, representing a reduction of approximately 20%. This verifies its efficacy in enhancing association robustness through penalties based on center distance and aspect ratio when addressing target deformation and partial occlusion. When both improvements are applied together, MOTA reaches 80.9%, and IDF1 achieves 83.7%, indicating increases of 4.1 and 3.5 percentage points, respectively, over the OC-SORT baseline. The number of IDs decreases from 18 to 12, corresponding to a reduction of about 33%, while Frag decreases from 15 to 10, also reflecting a 33% reduction. This synergistic enhancement arises from the complementary effects of the more accurate motion priors provided by the Transformer predictor and the enhanced occlusion robustness afforded by the BIOU metric, collectively yielding significant improvements in tracking continuity and identity preservation capabilities.

### 3.3. Parameter Setting and Sensitivity Analysis

To determine the optimal values of two key hyperparameters in the algorithm—the BIOU dynamic matching threshold *τ_biou_* and the second-stage fusion weight *ω*—parameter sensitivity experiments were conducted on the validation set.

(1) BIOU dynamic matching threshold *τ_biou_*

The parameter *τ_biou_* controls the strictness of BIOU matching in the initial stage. To identify the optimal threshold for various scenarios, *τ_biou_* was systematically scanned from 0.10 to 0.45 in increments of 0.05 across four distinct scenario subsets, utilizing MOTA as the evaluation metric. The results are presented in Table 6 below.

As shown in Table 6, the optimal value of *τ_biou_* demonstrates a significant dependency on the scenario: the optimal threshold is 0.30 for daytime and occluded scenes, while it decreases to 0.20 for nighttime and turbid water conditions. This variation reflects the differing detection quality across scenarios. In conditions with sufficient daylight and clear targets, accurate bounding box localization allows for the adoption of the 0.30 threshold to ensure matching precision. Conversely, in low-illumination nighttime conditions and turbid waters, where detection confidence diminishes and localization noise increases, lowering the threshold to 0.20 accommodates more uncertain association matches. This adjustment helps prevent trajectory interruptions that could arise from the premature rejection of low-quality detections.

(2) Fusion weight ω

In the second stage, the fusion weight *ω* between appearance similarity *S_a_* and motion similarity *S_m_* determines the relative contribution of these two features. A scan of *ω* from 0.1 to 0.9 (with a step size of 0.1) was performed on the validation set, using MOTA and IDF1 as evaluation metrics. The results are shown in Table 7 below.

As illustrated in Table 7, the performance remains stable and superior to extreme values when *ω* ranges between 0.6 and 0.8, with the optimal value identified at *ω* = 0.7. When *ω* is set to 0.1, the Multiple Object Tracking Accuracy (MOTA) declines to 78.2%, and at *ω* = 0.9, MOTA further decreases to 79.3%. This indicates that appearance and motion features are complementary, with appearance features contributing slightly more than motion features to data association. In the current experiments, *ω* is consistently applied as a fixed parameter across all scenarios.

(3) Historical trajectory length L

The length of the historical trajectory, denoted as *L*, determines the temporal context range accessible to the TrajectoryTransformer predictor. To identify the optimal value of *L*, we conducted comparative experiments on the validation set using L values of 5, 10, 20, and 30. The evaluation metrics employed included the Average Euclidean Distance (AED) between the predicted bounding boxes and the ground truth bounding boxes, as well as the Multiple Object Tracking Accuracy (MOTA). The results are presented in Table 8.

As shown in Table 8, when *L* increases from 5 to 10, the AED decreases from 0.0231 to 0.0196 while MOTA rises from 79.6% to 80.9%, indicating that the 0.17 s historical window is too short to capture the motion patterns of giant salamanders. When *L* increases to 20, the performance remains relatively stable, while an excessively long historical window (*L* = 30) leads to a slight performance decline. Considering both prediction accuracy and computational efficiency, this study selects *L* = 10 as the historical trajectory length.

### 3.4. Reliability Verification of Behavior Classification Rules

To evaluate the accuracy of behavior classification rules based on trajectory motion features, approximately 30 min video clips were randomly selected from each of the four scenario subsets in the test set: daytime, nighttime, turbid water, and frequent occlusion. Two trained observers independently conducted frame-by-frame manual behavior annotation. The consistency of inter-observer annotations was assessed using Cohen’s Kappa coefficient, which yielded a Kappa value of 0.87, indicating a strong agreement in the manual annotations.

The regularized decision method was applied to the tracked trajectories of Chinese giant salamanders in each video segment previously mentioned, utilizing a frame-by-frame comparison against manually annotated ground truth. The evaluation metrics employed include classification accuracy, precision, and recall, with the results presented in Table 9.

As shown in Table 9, the overall classification accuracy of the rule-based judgment method reaches 89.7%, with F1 scores exceeding 79% across all categories. Among these categories, the recognition precision is highest for resting behavior, followed by swimming and breathing; however, foraging behavior exhibits slightly lower accuracy. This discrepancy is primarily due to the overlapping motion characteristics shared between foraging behavior and slow swimming. The rule-based judgment method demonstrates strong reliability and can effectively support subsequent large-scale analyses of diurnal behavioral rhythms based on complete video trajectories.

### 3.5. Multi-Object Tracking Visualization Results

**Figure 9:**
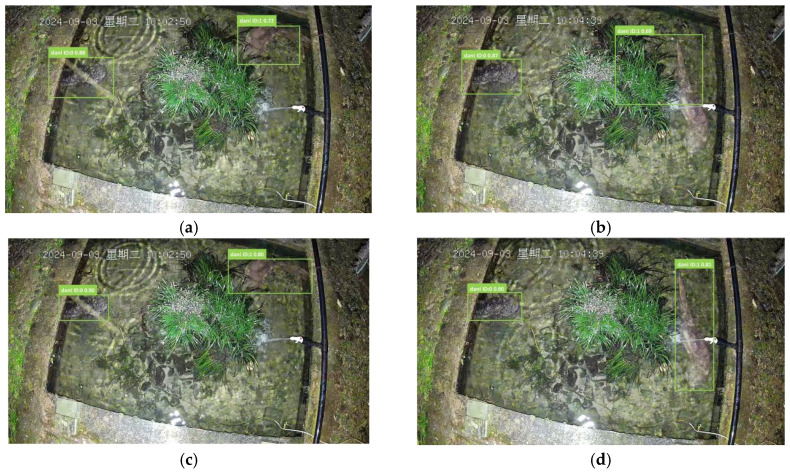
Visualization of the Transformer module. (**a**) Before Kalman filter module detection. (**b**) After detection by the Kalman filter module. (**c**) Before Transformer module detection. (**d**) After Transformer module detection. Note: The non-English term “星期二” in the figure translates to “Tuesday”.

To visually compare the performance of different motion prediction models in actual tracking, we selected consecutive frames that capture posture and direction changes during the swimming process of giant salamanders. Figure 9a,b illustrates the tracking results obtained using traditional Kalman filters. As demonstrated, when the targets (ID:0 and ID:1) commenced swimming, the predicted trajectories based on the linear constant velocity assumption of Kalman filters failed to align with the actual motion paths of the targets, resulting in significant deviations between the predicted and true detection boxes. This deviation was directly reflected in the decline of tracking confidence; for instance, the confidence level of ID:1 fluctuated markedly from 0.88 to 0.69, indicating the inadequacy of linear models in handling nonlinear motions, which can easily lead to target loss or trajectory drift. In contrast, Figure 9c,d present the results obtained using the Transformer predictor proposed in this study. Our model encodes both geometric and motion features of historical trajectories through a dual-branch architecture, enabling more accurate predictions of the swimming direction and position of giant salamanders. As shown in Figure 9b,d, the tracking confidence levels for both targets (ID:0 stabilized at 0.90, and ID:1 remained around 0.81) exhibited high stability throughout the entire movement process. This demonstrates that the Transformer module successfully learned and simulated the complex movement patterns of giant salamanders by capturing long-term dependencies in sequences, thereby providing more precise and reliable motion priors. Consequently, it significantly enhanced the robustness of subsequent data association and the overall stability of the tracking system.

**Figure 10:**
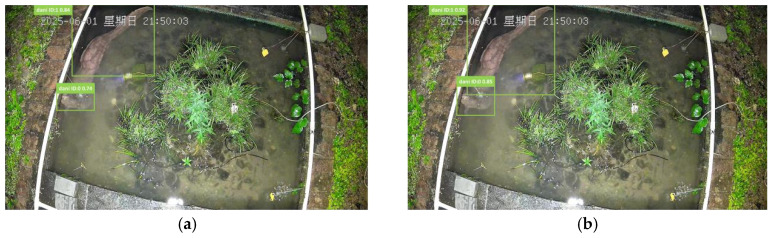
Visualization effect of BIOU module. (**a**) IoU module. (**b**) BIOU module. Note: The non-English term “星期日” in the figure translates to “Sunday”.

To evaluate the impact of association metrics on target localization accuracy, we compared the actual bounding box performance between traditional Intersection over Union (IoU) and the proposed Bounding Intersection over Union (BIOU) in complex underwater scenarios. Figure 10a presents the association results using standard IoU. As illustrated, the similar coloration between giant salamanders and the background leads to blurred detection edges. Coupled with potential deformations from their non-rigid bodies, the IoU metric, which relies solely on overlap area as an evaluation criterion, performs poorly. The generated tracking boxes (e.g., ID:0 and ID:1) exhibited significant deviations from the target’s true contours, either failing to fit tightly or displaying positional offsets. This primarily occurs because when overlap areas are similar, IoU cannot discern subtle advantages in position and shape between detection boxes. In contrast, the results using the BIOU metric, shown in Figure 10b, demonstrate its core advantage of multi-dimensional geometric evaluation capability. BIOU not only considers the overlapping area but also simultaneously assesses the distance between center points and the consistency of width-to-height ratios between predicted and detected bounding boxes. As illustrated in the figure, BIOU can select spatially more coherent bounding boxes that better match the target’s state in the previous frame from multiple possible detection candidates, thereby achieving more precise and stable target selection. This validates that the improvements in BIOU, designed to address common localization ambiguity and deformation issues in giant salamander tracking, are effective, directly enhancing the visual localization accuracy of tracking.

### 3.6. Performance Comparison in Complex Underwater Scenarios

To evaluate the algorithm’s adaptability in various challenging environments, we conducted performance analyses specific to different scenarios on the top-performing algorithm OC-SORT (excluding TransTrack-OC-SORT) and the baseline algorithm SORT across four subsets: daytime, nighttime, turbid water, and frequent occlusion. As shown in Table 10, TransTrack-OC-SORT maintains superior performance across all scenarios. In nighttime and turbid scenarios, it achieves MOTA scores of 75.8% and 74.1%, respectively, surpassing OC-SORT by over 5 percentage points. This advantage primarily arises from the Transformer’s ability to predict complex motion patterns and the BIOU’s tolerant matching mechanism for low-quality detection boxes. In scenarios with frequent occlusions, the algorithm’s trajectory fragmentation count (Frag) decreased to 18, representing a nearly 60% reduction compared to SORT. This demonstrates that the BIOU metric and the three-stage matching strategy can effectively maintain trajectory continuity during occlusions. In conclusion, the proposed algorithm exhibits excellent robustness across various complex hydrological and lighting conditions, fulfilling the practical requirements for all-weather monitoring of giant salamanders.

### 3.7. Comparison of Computational Efficiency

To evaluate the computational overhead of each algorithm in practical deployment, we compared the inference speed and GPU memory usage of TransTrack-OC-SORT with baseline algorithms on the same hardware platform (NVIDIA GeForce RTX 4070, PyTorch 1.10). The results are presented in Table 11.

As demonstrated in Table 11, TransTrack-OC-SORT achieves an inference speed of 31.6 FPS. Although this speed is lower than that of OC-SORT and SORT, it still surpasses the video capture frame rate of 30 FPS, thereby satisfying real-time processing requirements. The additional computational overhead is primarily attributed to the sequence inference of the dual-branch Transformer predictor and the motion feature extraction in the second stage.

### 3.8. Diurnal and Nocturnal Behavior Analysis of Giant Salamander Based on TransTrack-OC-SORT

**Figure 11:**
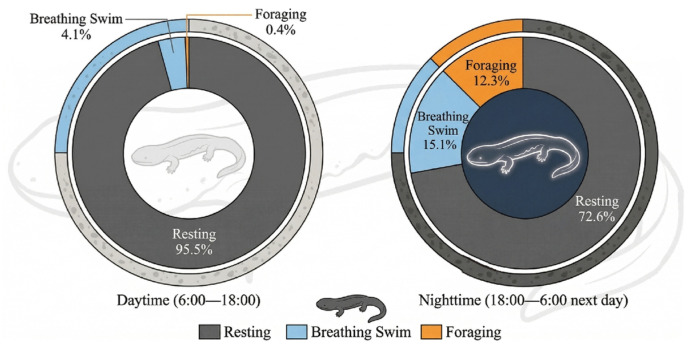
Behavior Statistics of Chinese Giant Salamander.

Based on behavioral classification rules, the tracking trajectories output by TransTrack-OC-SORT across all test videos were categorized into distinct behavioral types. This categorization yielded statistical results regarding behavioral distributions for two time periods: daytime (06:00–18:00) and nighttime (18:00–06:00 the following day), as illustrated in Figure 11.

The quantitative statistics and comparative analysis of behaviors during daytime and nighttime periods revealed fundamental differences in the behavioral patterns of the Chinese giant salamander, as illustrated in Figure 10. During daylight hours, the salamander exhibited pronounced sedentary tendencies, with stationary behavior accounting for 95.5% of observed activities, while swimming for respiration and foraging activities were minimal at 4.1% and 0.4%, respectively. In contrast, nighttime brought significant behavioral changes; although stationary behavior remained predominant, its proportion decreased substantially, accompanied by notable increases in both swimming for respiration and foraging activities.

## 4. Discussion

### 4.1. Results Analysis and Mechanism Discussion

The experimental results demonstrate that the TransTrack-OC-SORT algorithm significantly outperforms mainstream methods such as SORT, DeepSORT, ByteTrack, and OC-SORT. This superiority arises from its capacity to address two major challenges encountered by traditional methods in underwater environments. First, the dual-branch Transformer motion predictor effectively models the nonlinear motion patterns of giant salamanders, including variable-speed movements and turns, through dual-stream encoding of historical trajectory geometry and motion features. This approach resolves the model mismatch issue inherent in linear Kalman filtering in such scenarios. Second, the BIOU association metric introduces biologically inspired penalty terms for center distance and aspect ratio based on prior knowledge, thereby enhancing the traditional IoU to improve robustness in object detection. The results of the ablation study quantitatively support this assertion. The combination of these two enhancements leads to increased MOTA and IDF1 scores, along with significant reductions in both identity switches (IDs) and trajectory fragmentations (Frag). This establishes the necessity and complementary nature of the Transformer predictor design and the BIOU metric design.

In comparison to existing methods, SORT and DeepSORT rely on constant velocity models, making them susceptible to trajectory drift when confronted with the explosive movements of giant salamanders. Although ByteTrack and OC-SORT have optimized matching strategies, they do not fundamentally resolve the issue of nonlinear motion modeling. The Transformer predictor utilized in this study effectively captures long-range dependencies within sequences, facilitating more accurate predictions during sudden accelerations or sharp turns of targets. This results in particularly significant performance improvements in challenging conditions, such as nighttime or turbid water. These findings suggest that the integration of deep learning sequence models into wildlife tracking tasks for specific scenarios represents an effective strategy to overcome the performance limitations of traditional filtering methods.

### 4.2. Discovery of Behavioral Rhythms and Their Ecological Significance

Based on continuous trajectory data obtained from TransTrack-OC-SORT, this study quantitatively reveals significant diurnal behavioral rhythms in the Chinese giant salamander. Stationary behavior accounts for up to 95.5% during the daytime, while nocturnal surfacing and foraging behaviors increase significantly to 15.1% and 12.3%, respectively. These findings align closely with qualitative descriptions of the salamander’s nocturnal habits found in the literature [25,26], which were primarily derived from radio frequency identification technology and manual field observations. In contrast, this study offers continuous, systematic, and quantifiable data support through high-precision, non-invasive visual tracking. Compared to other amphibians, this prolonged pattern of daytime immobility resembles that of many nocturnal anurans [27,28]. However, the exceptionally high proportion of immobility reflects a unique behavioral strategy employed by large aquatic urodeles to mitigate predation risk and conserve energy.

From an ecophysiological perspective, the formation of this behavioral pattern arises from the interplay of multiple adaptive mechanisms. Firstly, regarding visual adaptation, the retina of the giant salamander is predominantly composed of rod cells, which are sensitive to low light but exhibit significantly reduced resolution in bright conditions [29,30]. Its daytime quiescence in rock crevices or caves serves as an adaptive strategy to mitigate visual disadvantages and decrease predation risks. Secondly, from the standpoint of energy metabolism, as a typical ambush predator, the giant salamander demonstrates a markedly decreased basal metabolic rate during periods of quiescence. Prolonged immobility during the day can be viewed as a behavior aimed at energy conservation, allowing for the accumulation of sufficient energy reserves for nocturnal foraging and territorial patrolling. Finally, from the perspective of predation ecology, the primary prey of the Chinese giant salamander—such as shrimp, crabs, and small fish—are predominantly crepuscular or nocturnal species [31,32,33]. Its nocturnal foraging behavior is closely synchronized with the active periods of its prey, thereby enhancing hunting success. The increased frequency of surfacing for air at night is directly linked to heightened oxygen demands during elevated activity levels, reflecting an adaptation in which physiological needs are aligned with behavioral patterns.

A small number of other sympatric species, such as fish and shrimp, were observed in the video recordings during this study. However, due to their significantly smaller body size, which is much smaller than that of juvenile giant salamanders, and their distinctly different movement behaviors, the few detection boxes generated by these animals were naturally categorized as independent short trajectories during tracking. This categorization occurred because of the substantial differences in appearance features and motion patterns, which did not interfere with the maintenance of giant salamander trajectories. The YOLOv11-based detector utilized in this study was trained on a giant salamander-specific dataset [34], with its detection primarily targeting giant salamanders. Additionally, the aspect ratio prior in BIOU further filtered out false detections with incompatible shapes at the geometric level. In the future, incorporating identification modules for other aquatic organisms into this system is expected to facilitate behavioral studies on interspecies relationships among multiple species.

### 4.3. Limitations and Future Perspectives

This study has certain limitations. Compared to traditional Kalman filtering, the dual-branch Transformer model incurs increased computational overhead, resulting in a reduction in the inference speed to 31.6 FPS. While this speed still meets real-time processing requirements on GPU-equipped workstations, deployment on other computing devices necessitates the application of model lightweighting techniques. Furthermore, the behavioral data analysis in this study emphasizes overall rhythm statistics and does not yet explore individual behavioral differences and social interactions.

Future research could explore several promising directions: (1) Investigating lightweight Transformer variants or knowledge distillation techniques to alleviate the computational burden of the model, thereby enhancing its suitability for real-time unattended monitoring systems in natural environments; (2) Implementing cross-scene domain adaptation methods to improve the algorithm’s generalization capability across various rivers, seasons, and lighting conditions; (3) Further integrating individual identification with fine-grained behavior classification modules to develop behavior profiles at the individual level; (4) Constructing social networks for Chinese giant salamanders at the individual level based on long-term accumulated trajectory data, which would provide more direct decision support for the refined management of protected areas.

## 5. Conclusions

This study addresses the challenges of nonlinear motion, drastic morphological changes, mimicry camouflage, and frequent occlusions in the multi-target tracking of giant salamanders in complex underwater environments by proposing the TransTrack-OC-SORT algorithm framework. The algorithm replaces traditional linear Kalman filtering with a dual-branch Transformer motion predictor, achieving precise modeling of irregular movement patterns, such as variable-speed swimming and directional changes in giant salamanders. Additionally, it introduces a BIOU association metric that incorporates biological constraints, combining center distance and aspect ratio penalties with geometric overlap to effectively enhance association robustness in occlusion and low-contrast scenarios. In the underwater tracking dataset of giant salamanders, TransTrack-OC-SORT achieved a multi-object tracking accuracy of 80.9%, an identity preservation metric of 83.7%, and only 12 identity switches. All metrics significantly outperformed existing mainstream algorithms, such as SORT, DeepSORT, ByteTrack, and OC-SORT. Ablation experiments confirmed the synergistic enhancement effect between the Transformer predictor and the BIOU metric, with their combined use improving MOTA by 4.1% and IDF1 by 3.5%. Based on the continuous trajectory data obtained by this algorithm, this study quantitatively revealed the diurnal behavioral rhythms of giant salamanders, thereby deepening the understanding of their nocturnal ecological habits from the perspectives of visual adaptation, energy allocation, and predation strategies. At the application level, TransTrack-OC-SORT provides a reliable, non-invasive technical solution for long-term behavioral monitoring in giant salamander reserves. Its core modules exhibit excellent transferability and can be extended to other aquatic or semi-aquatic species facing similar monitoring challenges after recalibration. Future work will focus on model lightweighting and enhancing cross-scenario generalization capabilities to better meet the practical requirements of long-term field deployment.

## Figures and Tables

**Table 1:** Camera Configuration.

Configuration Item	Detailed Description
Model	TL-PC6128-EZ
resolution	1200 pixels (4512 × 2512)
Night vision technology	Quad-pixel binning for enhanced low-light performance
Wireless support	Dual-band Wi-Fi (2.4/5 GHz)
Zoom	3× digital zoom
Protection level	IP66

**Table 2:** Data Collection.

Video Sequence Number	Scene	Video Duration/s	Purpose
1	Nighttime	1803	Training/Validation
2	turbid water	1704	Training/Validation
3	daytime	1143	Training/Validation
4	frequent occlusion	972	Training/Validation

**Table 3:** Performance Comparison of Algorithms.

Method	MOTA (%)	IDF1 (%)	HOTA	IDs	Frag	FP	FN
SORT	68.2	70.5	0.58	42	35	120	180
DeepSORT	72.1	75.3	0.62	28	22	95	150
ByteTrack	74.5	78.6	0.65	25	20	85	135
OC-SORT	76.8	80.2	0.68	18	15	70	120
TransTrack-OC-SORT	80.9	83.7	0.72	12	10	55	95

**Table 4:** Performance Comparison of General IoU Variants.

IoU Type	MOTA (%)	IDF1 (%)	IDs	Frag
IoU	70.5	73.2	35	31
GIoU	72.1	74.8	28	27
DIoU	73.8	76.1	22	23
CIoU	74.5	76.9	20	21
BIoU	80.9	83.7	12	10

**Table 5:** Ablation Experiment Results.

OC-SORT	Transform	BIOU	MOTA (%)	IDF1 (%)	HOTA	IDs	Frag
√	×	×	76.8	80.2	0.68	18	15
√	√	×	78.5	82.1	0.7	16	13
√	×	√	78.0	81.5	0.69	17	12
√	√	√	80.9	83.7	0.72	12	10

Note: √ indicates adoption of this method, and × indicates non-adoption of this method.

**Table 6:** Sensitivity Analysis of Dynamic Matching Threshold *τ_biou_* in Different Scenarios.

*τ_biou_*	Daytime MOTA (%)	Nighttime MOTA (%)	Turbid MOTA (%)	Occlusion MOTA (%)
0.10	76.2	72.5	71.3	68.9
0.15	77.8	74.1	72.6	69.6
0.20	79.5	75.8	74.1	71.2
0.25	80.1	75.0	73.5	71.8
0.30	80.9	74.3	72.8	72.3
0.35	79.8	73.2	71.5	71.6
0.40	78.6	71.8	70.1	70.5
0.45	77.1	70.2	68.5	70.2

**Table 7:** Sensitivity analysis of fusion weight *ω*.

*ω*	MOTA (%)	IDF1 (%)
0.1	78.2	80.5
0.2	78.6	80.9
0.3	79.0	81.3
0.4	79.5	81.8
0.5	79.9	82.3
0.6	80.4	83.1
0.7	80.9	83.7
0.8	80.5	83.3
0.9	79.3	81.6

**Table 8:** Sensitivity analysis of historical trajectory length L.

L (Frame)	Time Window (s)	AED	MOTA (%)
5	0.17	0.0231	79.6
10	0.33	0.0196	80.9
20	0.67	0.0192	80.8
30	1.00	0.0204	80.1

**Table 9:** Consistency Verification between Regularized Behavior Classification and Manual Annotation.

Behavior Category	Precision (%)	Recall Rate (%)	F1 Score (%)
Lying in ambush	94.2	96.8	95.5
Swim-breathing	86.5	82.1	84.2
Foraging	81.3	78.6	79.9
Overall	89.7	89.7	89.7

**Table 10:** Comparison of MOTA(%)/IDF1(%)/Frag across different scenarios.

Method	Daytime	Nighttime	Turbid	Occlusion
SORT	62.8/70.5/35	61.3/68.1/32	62.1/64.8/35	58.5/62.3/42
OC-SORT	76.8/80.2/15	70.5/73.8/22	68.2/71.5/25	65.8/64.9/30
TransTrack-OC-SORT	80.9/83.7/10	75.8/79.2/14	74.1/77.6/16	72.3/75.9/18

**Table 11:** Comparison of Inference Efficiency Among Algorithms.

Algorithm	FPS	GPU Memory (MB)
SORT	156.3	412
DeepSORT	48.7	864
ByteTrack	138.5	436
OC-SORT	142.5	428
TransTrack-OC-SORT	31.6	1258

## Data Availability

The data underlying the results of this research report is publicly available.

## Abbreviations

The following abbreviations are used in this manuscript:

MOTA	Multiple Object Tracking Accuracy
IDF1	ID F1 Score
HOTA	Higher Order Tracking Accuracy
IDs	ID Switches
FP	False Positive
FN	False Negative
UAV	Unmanned Aerial Vehicle
VGT-MOT	Visibility-Guided Tracking for Multiple Object Tracking
MOT	Multiple Object Tracking
BIOU	Bounded-IoU
GIoU	Generalized IoU
DIoU	Distance-IoU
CIoU	Complete IoU
AED	Average Euclidean Distance
